# Reduced Salivary Gustin and Statherin in Long-COVID Cohort with Impaired Bitter Taste

**DOI:** 10.3390/jcm13226816

**Published:** 2024-11-13

**Authors:** Harika Chowdary, Naomi Riley, Parul Patel, Ana G. Gossweiler, Cordelia A. Running, Mythily Srinivasan

**Affiliations:** 1 Oral Pathology, Medicine and Radiology, Indiana University School of Dentistry, Indianapolis, IN 46202, USA; 2 Oral Health Research Institute, Indiana University School of Dentistry, Indianapolis, IN 46203, USA; 3 Department of Periodontics, Indiana University School of Dentistry, Indianapolis, IN 46203, USA; 4 Department of Nutrition Science, Purdue University, West Lafayette, IN 47907, USA; crunning@purdue.edu

**Keywords:** long-COVID, saliva, taste, gustin, epithelial cells

## Abstract

**Background/Objectives**: Taste dysfunction is a frequent symptom of acute coronavirus disease (COVID)-19 caused by the severe acute respiratory syndrome coronavirus-2 (SARS-CoV-2). While the majority of those affected reported recovery over time, emerging data suggest that 20–25% of individuals experience persistent taste dysfunction, constituting a common symptom of long COVID. Gustation is mediated by continuously renewing taste bud cells. A balance between the counteracting processes of cell generation and cell death maintains the homeostatic turnover. Sonic hedgehog (SHH) is a morphogenic protein that promotes taste cell proliferation and differentiation. Enzymatic proteins such as gustin modulate the environment around the taste receptors and influence taste perception. Hence, we hypothesized that increased taste cell turnover and reduced taste-related salivary proteins contribute to the taste dysfunction in long COVID. **Methods**: Unstimulated whole saliva (UWS) was collected from individuals with long COVID experiencing taste dysfunction after obtaining informed consent. The normal control included archived saliva samples catalogued prior to 2019. Taste perception was objectively determined by the waterless empirical taste test. The SHH, gustin, and inflammatory cytokines in UWS were determined with ELISA. The expressions of epithelial and taste-cell-specific markers in cellular saliva were assessed by immunoflurorescence. **Results**: Impaired bitter taste was the most common dysfunction in the long-COVID cohort. Salivary gustin was significantly lower in those with long COVID and correlated with lower bitter taste score. Cellular saliva showed keratin-10- and small-proline-rich protein-positive epithelial cells as well as SHH-, occluding- and KCNQ1-positive taste cells. **Conclusions**: Salivary gustin could be a marker for impaired bitter taste in long COVID.

## 1. Introduction

A significant proportion of COVID-19 survivors experience lingering and debilitating symptoms following acute COVID-19 infection. Amongst the multitudes of symptoms reported in long COVID, multiple studies suggest that anosmia and dysgeusia are frequent symptoms of long COVID [1,2,3].

Multiple studies reported smell and taste dysfunction as relatively frequent symptoms of long COVID [1,2,3]. The striking features of chemosensory disturbances in long COVID are the persistence for long periods and phantom tastes. Taste perception is mediated by the gustatory receptor cells in the taste buds innervated by the facial, glossopharyngeal, and vagus nerves. Specialized receptor cells innervated by the olfactory nerve in the nasal epithelium mediate the sense of smell. Comprehensive perception of flavor is mediated by the multimodal interaction between the sensations of smell, taste, and chemesthesis as retronasal olfaction is a major contributor to flavor while eating [4,5,6]. Hence, differentiation between taste dysfunction from loss of smell is often very difficult.

Several reasons have been postulated to account for the taste disturbances in long COVID. Evidence of CoV-2 virus and inflammatory infiltrates in post mortem tongue tissues of patients with COVID-19 support the direct invasion and inflammation as causative of dysgeusia. Consistently, oral epithelial cells including the taste cells have been shown to exhibit angiotensin converting enzyme-2 (ACE-2), the primary receptor for CoV-2, and the presence of the virus in post mortem tongue tissues. A soluble form of ACE2 (sACE2) is released constitutively and is increased by inflammation [7]. Higher concentrations of circulating sACE2 have been reported in human pathologies such as asthma and chronic kidney disease, with increased propensity for infection [8,9,10]. The salivary level of sACE2 has been reported to be higher than that in plasma [7]. We observed that the sACE2 level was higher in the saliva of patients with COVID-19 and correlated strongly with the viral load [11].

The lingual epithelium is covered by “tongue film”, composed of exfoliated cells, residual saliva, and the microbiota. Hence, taste sensitivity is modulated by the cellular density and the bacterial microbiota in the tongue film [12,13]. Dysbiosis secondary to long viral infections, such as COVID-19, disrupts the commensal homeostasis and induces innate inflammatory responses, which result in increased epithelial proliferation and exfoliation [14,15,16]. As such, pressure to replace the exfoliated taste receptor cells by stem cells could disrupt and alter epithelial homeostasis, affecting taste perception [17].

Saliva plays an integral role in taste sensitivity as a conduit of substances to taste receptors that triggers signal transduction, culminating in specific taste perception [18,19]. In taste dysfunction, dysbiosis-induced inflammation and disrupted epithelial homeostasis can be reflected in clarified and cellular saliva. In clarified saliva, sonic hedgehog (SHH) protein, known to contribute to the cellular turnover and homeostasis [20,21], and gustin or carbonic anhydrase-VI (CA-VI) enzymatic protein, which modulate the buffering potential in the vicinity of taste receptor cells, have been shown to contribute to appropriate response to various taste stimuli [22,23,24]. Furthermore, salivary-protein-associated dysbiosis also modulate taste sensitivity. Statherin is a low-molecular-weight phosphoprotein that contributes to biofilm formation by interacting with microbes and facilitating specific microbial adherence [25]. Since periodontitis is recognized as a comorbid condition with COVID-19, altered salivary statherin could negatively influence taste perception [26,27].

The aims of the present study were to (1) obtain objective measures of taste and smell scores in individuals with history of subjective symptoms of persistent loss of taste/smell following SARS-CoV-2 infection; (2) determine levels of salivary proteins involved in taste perception including the morphogenic protein SHH, enzymatic protein gustin, and statherin, a secondarily associated protein; (3) explore the associations between salivary proteins known to be involved in taste perception and the nature of taste disturbance in those after having COVID-19; and (4) investigate the effect of long COVID on salivary inflammatory cytokines. To the best of our knowledge, there is no report on chemosensory dysfunction and salivary proteins in the same cohort.

## 2. Materials and Methods

### 2.1. Study Design

Individuals with a history of a positive SARS-CoV-2 test and self-reported taste and/or smell disturbances were identified from amongst the responders to our previous survey report on the frequency of long COVID amongst those registered at IU School of Medicine’s COVID-19 Research Registry [28,29]. The responders reported the date of the initial positive testing and the number of times they tested positive subsequently. Individuals were recruited by phone and email from amongst these responders with self-reported taste disturbance in accordance with the institutional review board approval. Thirty subjects completed the objective taste and smell strip tests. The demographic information is given in Table 1.

### 2.2. Taste Test Administration

Taste function was assessed using a validated waterless empirical taste test (WETT) [30]. Test items consisted of 53 paper taste strips, with a 1 × 2.5 cm monomer cellulose pad that contained a concentration of dried sucrose (0.20, 0.10, 0.05, or 0.025 g/mL), citric acid (0.025, 0.05, 0.10, or 0.20 g/mL), sodium chloride (NaCl; 0.0313, 0.0625, 0.125, or 0.25 g/mL), caffeine (0.011, 0.022, 0.044, or 0.088 g/mL), monosodium glutamate (MSG; 0.017, 0.034, 0.068, or 0.135 g/mL), or no stimulus. Each participant was instructed to move the cellulose pad of the strip around the mouth, particularly along the tip and edges of the tongue for 5 to 10 s and to identify the taste quality from the 5 categories listed on the response form (sweet, sour, salty, bitter, and brothy) or to indicate that no taste could be perceived. The WETT presents each stimulus from weak to strong concentration in an ascending sequence in the first half and in reverse presentation, from strong to weak concentrations in the second half. Different tastants were presented in a random order except that no tastant immediately followed itself. Blanks were interspersed after the higher concentrations of caffeine, NaCl, and citric acid to remove the residual stimulus. The WETT is highly reliable, with test–retest and split-half reliability coefficients of 0.92 and 0.88, respectively [31].

### 2.3. Sense of Smell Test

Smell function was tested using the highly validated University of Pennsylvania Smell Identification Test (UPSIT) [32,33,34]. This is a 40-item forced-choice test assessing the comparative ability of an individual to identify odorants at the suprathreshold level. Each participant is given four envelope-size booklets, each containing 10 scratch and sniff odorants embedded in 10 to 50 μm microcapsules positioned on brown strips at the bottom of each page. Each strip has a multiple-choice question with four alternatives. The test score is the number of correct responses for 40 odorants.

### 2.4. Saliva Collection and Isolation of Epithelial Cells

Unstimulated whole saliva (UWS) was collected as previously described [35]. Briefly, participants were asked to refrain from eating or drinking for 1 h prior to saliva collection. At least 1 mL of UWS was collected by passive drooling for 10 min into a prechilled centrifuge tube. The UWS flow rate ranged between 0.3 mL/min and 0.6 mL/min. All UWS samples were centrifuged at 250× *g* for 10 min at 4 °C. The supernatant-clarified saliva was collected, to which we added a protease inhibitor cocktail solution (Roche Diagnostics, Indianapolis, IN, USA) in 1:1 *v*/*v* ratio and stored at −80 °C until future analysis. The cellular sediment was resuspended in isotonic saline in a 1:10 volumetric ratio, two drops of Zap-O-globin were added to lyse the blood corpuscles, which were then centrifuged at 1271.7× *g* for 10 min at 4 °C. The cell suspension was then filtered through a membrane with a 20 micron pore size. The membrane-trapped salivary epithelial cell (SEC)-enriched preparation was assessed by light microscopy for appropriate morphology, reconstituted in RPMI-1640 (Mediatech Inc., Minnesota, MN, USA), supplemented with 5% fetal bovine serum (Hyclone fetal bovine serum, Thermo Scientific, Logan, UT, USA) and 5% dimethylsulfoxide (Sigma-Aldrich, St Louis, MO, USA), and stored at −80 °C until further analysis.

### 2.5. Immunofluorescence Staining of SEC

Immunofluorescence was performed using primary antibodies against pan cytokeratin (1:100; pan-Cytokeratin Antibody (AE1/AE3): sc-81714, Santa Cruz Biotechnology, Inc.; Dallas, TX, USA), SHH (Catalog #: AF464, R&D Systems, Minneapolis, MN, USA), and occludin (1:250: sc-133256, Santa Cruz Biotechnology, Inc.; Dallas, TX, USA). Briefly, SECs were fixed at −20 °C for 20 min using absolute methanol and were permeabilized using a 0.3% solution of Triton in methanol. This was followed by blocking with 5% fetal bovine serum for 1 h at room temperature. Furthermore, the cells were incubated with the primary antibodies overnight at 4 °C, followed by incubation with Alexa Fluor488 or Alex Flour555 conjugated secondary antibody (Jackson ImmunoResearch Laboratories, Inc, Wet Grove, PA, USA) for 1 h at room temperature. Nuclei were stained with DAPI (1:4000, BD Biosciences, San Diego, CA, USA). Immunostained cells were mounted with ProLong™ Gold antifade mounting medium (Thermofisher, Waltham, MA, USA). Images were recorded with a NIKON multiphoton microscope attached to a DS Ri2 Camera (NIKON Instruments Ind, Melville, NY, USA).

### 2.6. ELISA for sACE2 and Anti-ACE2 IgG

The sACE2 in saliva was measured with a human ACE-2 sandwich ELISA kit (Invitrogen, Thermofisher Scientific, Waltham, MA, USA) [11]. Normal or control samples were obtained from archived UWS stored in a saliva biobank prior to 2019. We previously showed the well-preserved quality of the archived samples in our saliva bank for assessing protein and genetic markers [35]. High-binding plates (Corning Inc., Corning, NY, USA) were coated with ACE2 recombinant protein (Cat# E80013-1, Epigentek, Farmingdale, NY, USA), diluted to 2 μg/mL in 0.1 M sodium bicarbonate, and incubated overnight at 4 °C. After washing, the coated wells were probed with UWS diluted to 1:50 and incubated at room temperature for two hours. The wells were washed and then incubated with biotinylated goat anti-human IgG (Cat# 109-036-098, Jackson ImmunoResearch, West Grove, PA, USA) within the range of the manufacturer’s recommendations as at IgG 1:50,000. Bound antibodies were detected using HRP-conjugated secondary antibody followed by TMB (3,3′,5,5′-tetramethylbenzidine) substrate (Pharmingen, San Diego, CA, USA). Absorbance was measured at 450 nm immediately after 0.33 N HCl stop solution was added to a microplate reader (Biorad Laboratories, Hercules, CA, USA).

### 2.7. Determination of Salivary SHH, Gustin, and Statherin

The levels of SHH protein, gustin, and statherin in UWS were determined using a Human Sonic Hedgehog ELISA Kit (ABIN6970368), Human Statherin ELISA Kit (ABIN6959710) and Human CA6 (Carbonic Anhydrase VI) ELISA Kit (ABIN5524151) (Antibodies.com LLC, St. Louis, MO, USA).

### 2.8. ELISA for Cytokines and sCD14

The protein content of the clarified saliva was determined by spectrophotometry using the Bradford method to increase the sensitivity of detection of low-abundance proteins. Each UWS sample was depleted of amylase by incubating serially with anti-human amylase mAb (1:2500; Abcam, Cambridge, MA, USA) and protein G beads (Miltenyi Biotec Inc, Auburn, CA, USA) at 4 °C. One microgram of protein from each treated UWS sample was assessed for the presence of sCD14, IL-6, IL-8, and IFN-γ using commercially available ELISA kits (R&D Systems, Minneapolis, MN, USA). Absorbance at 450 nm was measured with a microplate reader (Biorad Laboratories, Hercules, CA, USA).

### 2.9. Quantitative Real Time Polymerase Chain Reaction

The RNA isolated from SEC using Quick RNA microprep kit (Zymo Research, Irvine, CA, USA, cat#R1050) was reverse-transcribed using a cDNA Synthesis Kit (Catalog number: K1621, Thermo Fisher Scientific, Waltham, MA, USA). Real-time quantitative PRC (qPCR) was performed using SYBR™ Green PCR Master Mix (Catalog number: 4344463, Thermo Fisher Scientific, Waltham, MA, USA). The primers used for the analysis were as follows: for keratin 10 (K10), 5′-TTCTAGCAGCAAAGGCTCCC-3′ and 5′-CTGCCTCCATAACTCCCACC-3′; for TAS2R38 5′-GTGCTGCCTTCATCTCTGTGCC-3′ and 5′-GCTCTCCTCAACTTGGCATTGC-3′; for β-actin-F: 5′-GCCAACCGCGAGAAGATGA-3, βactin-R: 5′-CATCACGATG CCAGTGGTA-3’. The fold change in expression was analyzed via the 2^−ΔΔ*CT*^ method using β-actin as the housekeeping gene [11].

### 2.10. Statistical Analysis

The WETT taste scores of the long-COVID cohort were compared to the normative data for the test obtained from the manufacturer. Differences between the long-COVID and pre-COVID-19 saliva were determined by Student’s *t*-test and post hoc analysis. The Wilcoxon rank sum test was used for non-parametric comparison of salivary gustin or SHH level with the bitter taste score. A 5% significance level was used for all tests.

## 3. Results

### 3.1. Participant Details and Objective Taste and Smell Scores

**Figure 1:**
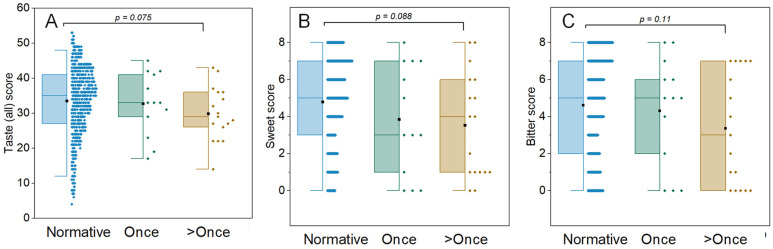
Long-COVID group display patterns tending toward lower scores compared to the normative data from the test manufacturer: (**A**) total taste score, (**B**) sweet score, and (**C**) bitter score.

The demographic features of the study subjects are provided in Table 1. Thirty participants completed objective taste and strip tests. Equivalent numbers of women and men reported one or two positive CoV-2 tests. The average age did not differ between the cohort of individuals with one positive CoV-2 test (56 ± 12.9 years) or more than one positive CoV-2 test (46.5 ± 11.6 years). A study dentist examined all participants. All study participants had good oral health with no other clinically identifiable lesion. Taste function was assessed objectively using the well-validated 53-item self-administered WETT test. By correlating subjective complaints with objective evaluations of chemosensory perception, we observed that the total taste score was significantly lower in individuals reporting taste perception being worse than before SARS-CoV-2 infection. However, the objective smell scores did not differ between individuals’ subjective complaints of sense of smell, being same as or worse than pre-COVID-19 (Table 1(A)). The mean total taste and smell scores of individuals with history of one positive COVID test or more than one positive COVID test were similar (Table 1(B)). Amongst the one positive COVID test cohort, bitter and sweet taste dysfunction (specific taste score of 0–1) was experienced by 26% and 16% of individuals, respectively. Amongst the cohort with more than one positive COVID test, nearly twice the number of individuals experienced impaired sweet taste perception (12%) as compared to those with impaired bitter taste (6%) (Figure 1).

### 3.2. Measures of sACE2 and Anti-ACE2 in Long COVID

**Figure 2:**
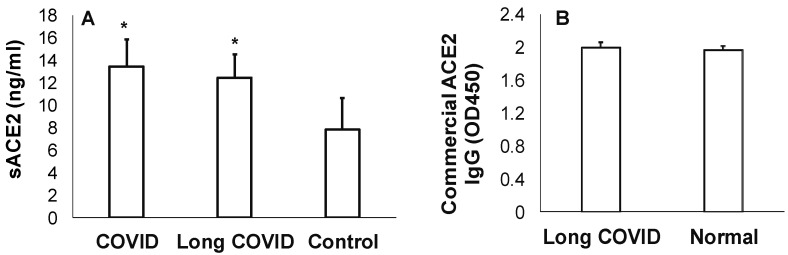
Salivary soluble ACE-2 level is elevated in long-COVID: UWS samples collected from our long-COVID cohort were assessed for (**A**) sACE2 and (**B**) anti-ACE2 IgG by ELISA as described in the Section 2. * = *p* < 0.05.

We observed that the sACE2 level was significantly higher in COVID-19 or long-COVID saliva samples than in pre-COVID-19 saliva samples (Figure 2A). Previously, sACE2 in saliva has been variably reported as elevated or lower in patients with COVID-19 [11,36]. All individuals in our long-COVID cohort had received COVID-19 vaccination and at least one booster. We observed no differences in the levels of anti-ACE2 antibody in the long-COVID and pre-COVID-19 saliva samples (Figure 2B). Previously, antibodies to ACE2 were found to be increased in individuals with CoV-2 [37].

### 3.3. Salivary SHH, Gustin, and Statherin in Long-COVID

**Figure 3:**
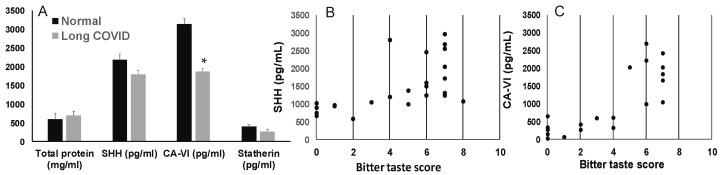
Measures of SHH, CA-VI, and statherin in long-COVID saliva: UWS samples collected from our long-COVID cohort were assessed for (**A**) SHH, CA-VI, and statherin by ELISA. * = *p* < 0.05. Wilcoxon signed rank test showed that the distribution of concentration of salivary SHH (**B**) and gustin (**C**) across the objective bitter score significantly differed (Z = −4.0156, *p* < 0.0001).

We observed that the salivary SHH was lower in the long COVID cohort as compared to the pre-COVID-19 saliva, although the difference was not statistically significant (Figure 2A). However, within the long-COVID cohort, salivary SHH was lower in individuals with impaired bitter taste or lower bitter taste scores of 0–2 (Figure 2B). We observed that the salivary gustin or CA-VI level was significantly lower in the long-COVID cohort as than in the pre-COVID-19 saliva (Figure 3A). Pertinently, individuals with loss of bitter taste (bitter taste score 0) had significantly lower CA-VI levels than those with high sensitivity to bitter taste (bitter taste score 7) (Figure 3B). Since periodontitis is recognized as a comorbid condition with COVID-19 and could negatively influence taste perception, we assessed salivary statherin [26,27]. Our data showed no significant differences in the salivary statherin levels between the long-COVID and the pre-COVID-19 saliva (Figure 3C).

### 3.4. Bitter Taste Receptor, T2R38, in Long COVID

#### 3.4.1. Epithelial Cells in Saliva Express SHH and Occludin

**Figure 4:**
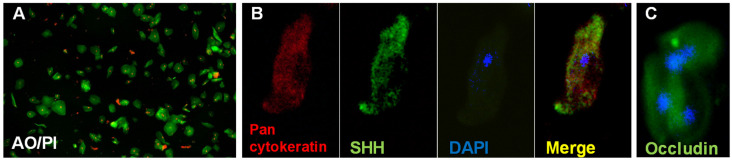
(**A**) Epithelial cells in saliva stained with AO/PI. Majority of the epithelial cells are viable as indicated by AO+ staining. (**B**) A representative image of cell in saliva staining positive for SHH and (**C**) occludin.

Taste receptor cells are maintained by the proliferation of stem cells, which replenishes loss by exfoliation [4,21]. We hypothesized that taste dysfunction could be related to the increased exfoliation of taste cells. The number of epithelial cells in saliva has been shown to vary between 0.1 million and 0.9 million cells per mL [35,38,39]. We observed that the number of epithelial cells in long-COVID saliva varied between 0.3 and 0.8 million cells/mL. Most epithelial cells were alive, as shown by the AO/PI staining (Figure 4A), which was positive for pan cytokeratin and some for SHH (B) and occludin (C).

#### 3.4.2. Expression of T2R38 and Cytokines in Long-COVID Saliva

**Figure 5:**
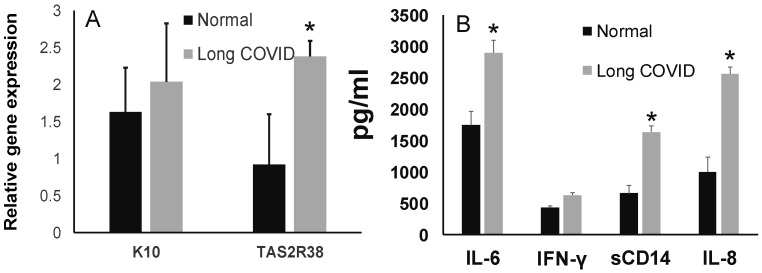
Expression of TAS2R38 and salivary cytokines in long COVID saliva. (**A**) The relative expression of keratin-10 and TAS2R38 mRNA was assessed by RT-PCR, and (**B**) the levels of cytokines in saliva were measured by ELISA. * = *p* < 0.05, significant.

Bitter taste receptors (T2Rs) are G protein-coupled receptors (GPCRs) involved in bitter taste perception on the tongue. Although 25 different T2Rs are known to be expressed in humans, polymorphism in the T2R38 gene has been widely studied with respect to bitter taste perception [40,41]. In our long-COVID cohort, we observed a similar distribution of the taster and nontaster haplotype as in the general population. However, we observed that the fold change in T2R38 was significantly higher in the long-COVID salivary epithelial cells than that in the cells in the pre-COVID-19 saliva (Figure 5A). Since taste receptor expression has been shown to be associated with inflammation [42], we measured the inflammatory cytokines in the saliva. We observed that the levels of the innate immune marker, sCD14, as well as cytokines IL-6, IL-8, and IFN-γ (Figure 5B), were significantly lower in the long-COVID saliva than in the pre-COVID-19 saliva.

## 4. Discussion

The major findings in the present study included the following: (1) Amongst the different taste sensitivities, impairment of bitter taste was more frequent in individuals previously infected with SARS- CoV-2. (2) While both salivary SHH and CA-6 levels were decreased in long COVID, the reduced CA-6 or gustin concentration correlated with a lower taste score. (3) The epithelial cells in long-COVID saliva exhibited elevated TAS2R38 transcript levels, and (4) inflammatory cytokines and innate-associated sCD14 levels were upregulated in the UWS of the long-COVID cohort.

The recent CoV-2 pandemic differs uniquely from the previous coronaviruses in exhibiting a higher prevalence of disturbances of taste, smell, and chemesthesis [43,44]. Furthermore, as opposed to the taste dysfunction in CoV1 and Middle Eastern Respiratory Syndrome (MERS) that was often reported with smell disturbances, observations in the recent CoV-2 pandemic suggest that the taste disturbances reflect actual impairment of gustatory abilities without olfactory dysfunction [45,46]. The emerging data suggest that the taste and smell disturbances persist for extended periods, constituting frequent observations in long-COVID syndrome [2,3,16].

Flavor perception is known to be mediated by the multimodal interactions between taste, smell, and chemesthesis. Hence, it is critical to investigate salivary parameters along with objective measures of taste and smell sensitivity to gain a better understanding of taste dysfunction. In our cohort of individuals with history of a positive COVID-19 test, while bitter taste distortion was the most prevalent, most of these same individuals also scored low on sweet perception. Other tastes were not affected significantly. This is consistent with other reports of taste dysfunction in long COVID [5,47].

The pathogenesis of the taste alterations in long COVID is not clear. It has been suggested that CoV-2 infection and inflammation induce epithelial damage, including the taste papillae, to interfere with the supply of nutrients or stimulants, resulting in the reduced replication and depletion of taste cells, contributing to taste dysfunction [4,12,15]. The extruding taste cells are increased in cellular saliva, and we observed the epithelial cells expressed markers of taste cells in long-COVID saliva.

SHH is a morphogenic protein secreted by the epithelial cells, including the taste receptor cells, and signaling via SHH has been shown to be involved in regulating taste cell homeostasis [21]. Viral infections such as by influenza have been known to interfere with SHH signaling, disrupt epithelial junctions, and consequently increase epithelial exfoliation [48]. The. presence of SHH-positive cells in long-COVID saliva suggests the presence of epithelial cells in the process of differentiation, since the disruption of SHH pathways has been shown to impair taste bud cell proliferation and differentiation, as well as interfere with taste perception [48,49]. Previously, SHH has been reported in both glandular and whole saliva [50,51]. Levels of salivary SHH has been associated with taste sensitivity with reduced levels reported in conditions of taste dysfunction [20,51]. Consistently, we observed SHH levels were reduced in the saliva of individuals with long-COVID as compared to those in pre-COVID-19 saliva.

Specific gene alleles have been associated with bitter taste perception. Polymorphism of the bitter receptor gene TAS2R38 has been identified to differ between tasters, supertasters, and non-tasters [41]. Gustin gene polymorphism has been shown to be important for discriminating the bitter taste of 6-n-propylthiouracil [23,24]. Furthermore, a lower CA-VI/gustin concentration in saliva has been associated with reduced bitter taste perception [22,23]. Consistent with these observations, our data also showed that the individuals with long COVID with a lower bitter score exhibited lower salivary concentrations of gustin.

In addition to taste perception, TAS2R38 signaling has been shown to induce innate immune responses in epithelial cells [40]. Consistently, we observed that the chemokines and innate-immune-associated sCD14 levels were elevated and that the relative expression of the TAS2R38 transcript was higher in long-COVID saliva. Inflammation, together with the impaired bitter taste and related changes in dietary habits, may contribute to the increase in new-onset diabetes post-COVID-19 [52,53]. Participants in our long-COVID cohort had good oral health, with salivary statherin levels comparable to those in control saliva.

**Figure 6:**
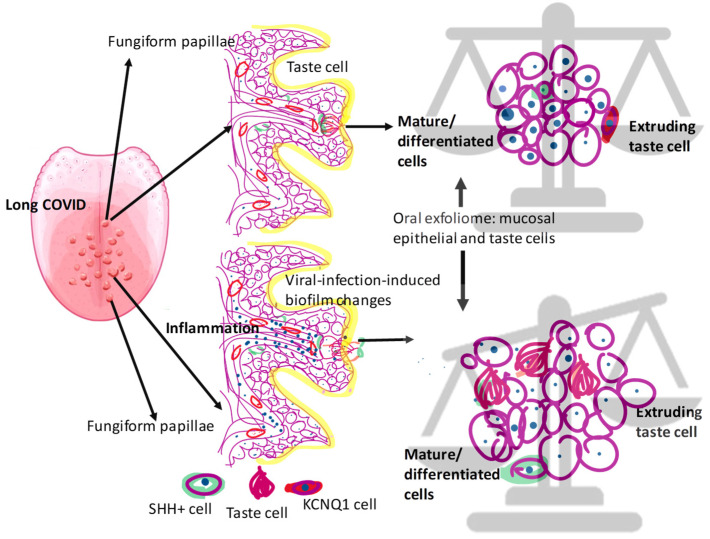
Schematic representation of suggested mechanism for taste dysfunction in long COVID. In healthy people, non-lingual epithelial cells and taste cells exhibit a turnover of 14–21 days and 8–10 days, respectively. These cells constitute the oral exfoliome. In long COVID, the changes induced in the biofilm following CoV-2 infection mediate chronic inflammation and disrupt the balance between taste and non-taste cell proliferation and exfoliation, shifting the balance towards increased taste cells in the exfoliome, consequently leading to taste dysfunction. Representative positive taste cell marker cells are shown.

Taken together, we project a model for the potential mechanism of taste dysfunction in long COVID (Figure 6). Viral persistence in tissues and the consequent chronic inflammation, even in the absence of active infection, disrupt the homeostasis between the proliferation and exfoliation of both taste and non-taste epithelial cells, shifting the balance towards increased loss, leading to taste dysfunction. Consequently, an increased number of cells in the process of maturation exhibiting SHH and cells exhibiting the neuronal markers of taste perception are present in the oral exfoliome, representing the epithelial cells in saliva.

### Limitations

Our study presents some limitations. First, the relatively small number of samples suggest that the results should be interpreted with caution. Second, we examined the relative frequency of altered taste associated with COVID-19 reinfection compared with that in a single infection. The results do not represent an assessment of severity of a second infection versus that of a first infection. Third, this study did not relate chemosensory dysfunction with the multiorgan symptoms typical of long COVID.

## 5. Conclusions

In conclusion, we report for the first time that the levels of taste-related proteins are lower in saliva in long COVID and that the reduction is related to an impaired perception of bitter taste. Our observation of bitter taste being the predominant taste affected in long COVID is in concurrence with the reported frequency of impaired bitter taste in previous studies. Furthermore, exploring whether variations in salivary SHH and CA6 influence taste sensitivity or preference could open new avenues for understanding the complexities of taste perception and its implications for health and disease.

## Figures and Tables

**Table 1:** (A) Participant characteristics and average total taste and smell score. (B) Concurrence of subjective symptoms and objective measures of taste and smell perception. (C) Distribution of the taste dysfunction in our cohort of individuals with history of one or more positive SARS-CoV-2 tests.

**(A) Subjective Complaints**	**Same as Pre-COVID-29**	**Worse After COVID-19**
Taste	16	14
Smell	18	12
Mean total taste score	33.7 ± 7.36	28.7 ± 8.8
Mean total smell score	36.2 ± 4.5	35.2 ± 4
**Objective Chemosensory Tests** **(B) # of Positive COVID-19 Tests**	**Once**	**>Once**
Number (male: female)	13 (6:7)	17 (8:9)
Age	56 ± 12.9	46.5 ± 11.6
Mean total taste score	31.2 ± 7.8	30.8 ± 9.1
Mean total smell score	35.3 ± 2.9	36.6 ± 2.2
**Objective Chemosensory Tests** **(C) # of Positive COVID-19 Tests**	**Once**	**>Once**
Number	13	17
Impaired bitter taste	16%	26%
Impaired sweet taste	6.50%	12.90%
Impaired salt taste	6.50%	3%
Impaired sour taste	6.50%	3%

## Data Availability

The data presented in this study are available upon request from the corresponding author. The data are not publicly available due to individual privacy and ethical reasons.
